# Nextmed: Automatic Imaging Segmentation, 3D Reconstruction, and 3D Model Visualization Platform Using Augmented and Virtual Reality

**DOI:** 10.3390/s20102962

**Published:** 2020-05-23

**Authors:** Santiago González Izard, Ramiro Sánchez Torres, Óscar Alonso Plaza, Juan Antonio Juanes Méndez, Francisco José García-Peñalvo

**Affiliations:** 1 ARSOFT: Salamanca, Castilla y León, 37008 Salamanca, Spain; 2 Department of Human Anatomy, University of Salamanca, 37008 Salamanca, Spain; ujhik@usal.es (R.S.T.); u157938@usal.es (Ó.A.P.); jajm@usal.es (J.A.J.M.); fgarcia@usal.es (F.J.G.-P.)

**Keywords:** augmented reality, virtual reality, medical imaging, automatic segmentation

## Abstract

The visualization of medical images with advanced techniques, such as augmented reality and virtual reality, represent a breakthrough for medical professionals. In contrast to more traditional visualization tools lacking 3D capabilities, these systems use the three available dimensions. To visualize medical images in 3D, the anatomical areas of interest must be segmented. Currently, manual segmentation, which is the most commonly used technique, and semi-automatic approaches can be time consuming because a doctor is required, making segmentation for each individual case unfeasible. Using new technologies, such as computer vision and artificial intelligence for segmentation algorithms and augmented and virtual reality for visualization techniques implementation, we designed a complete platform to solve this problem and allow medical professionals to work more frequently with anatomical 3D models obtained from medical imaging. As a result, the Nextmed project, due to the different implemented software applications, permits the importation of digital imaging and communication on medicine (dicom) images on a secure cloud platform and the automatic segmentation of certain anatomical structures with new algorithms that improve upon the current research results. A 3D mesh of the segmented structure is then automatically generated that can be printed in 3D or visualized using both augmented and virtual reality, with the designed software systems. The Nextmed project is unique, as it covers the whole process from uploading dicom images to automatic segmentation, 3D reconstruction, 3D visualization, and manipulation using augmented and virtual reality. There are many researches about application of augmented and virtual reality for medical image 3D visualization; however, they are not automated platforms. Although some other anatomical structures can be studied, we focused on one case: a lung study. Analyzing the application of the platform to more than 1000 dicom images and studying the results with medical specialists, we concluded that the installation of this system in hospitals would provide a considerable improvement as a tool for medical image visualization.

## 1. Introduction

Although medical imaging technology has continued to evolve over the last decades, the technology used for visualization and analytical purposes has remained constant since the appearance of workstations. However, several tools (OsiriX, 3DSlicer) are capable of semi-automatically producing high resolution 3D pictures from these medical images in a few seconds [1,2]. This manual or semiautomatic segmentation is, in many cases, complicated and requires the intervention of a specialist [3,4]. Thus, the automatic segmentation of anatomical structures is of considerable interest to medical practitioners, as it allows them to immediately start working, using 3D models that can facilitate clinical diagnoses. Although much research has been conducted on automatic segmentation [5,6,7,8,9,10,11,12], the use of a complete system capable of automatic segmentation on a large scale in hospitals, using traditional computers and unspecialized workstations and allowing augmented reality (AR) and virtual reality (VR) visualization, has not yet been achieved. Addressing this situation and providing an innovative visualization tool were the main objectives of this work and the result led to the Nextmed project [13,14].

In comparison with the standard technology used for 3D visualization of medical images, AR and VR provide us with a 3D visualization system that is more realistic, allowing doctors to effectively interact with the generated 3D model and facilitating pre-surgical study [15,16].

The aim of artificial intelligence research is to train algorithms that allow segmenting without incurring possible errors that contain manually programmed artificial vision algorithms [17,18,19]. One of the most widely used techniques for attempting automatic segmentation using artificial intelligence involves convolutional neural networks (CNNs) [11]. The significant advances in recent years have resulted in neural networks [20] that can be trained to not only recognize parts of an image, but also to generate a tagged binary mask, such as (regions with convolutional neural network) [17,21], U-Net [22,23], V-Net [18,24], SegAN [25], and others for specific regions, such as for pancreas segmentation [26]. All these neural networks can be included in the Nextmed platform to increase the number of anatomical regions that can be automatically segmented.

The problem is obtaining enough tagged data that allow the artificial intelligence (AI) algorithms to be trained. For this reason, we implemented artificial vision algorithms.

Regarding visualization technologies, augmented and virtual reality have quickly improved during the recent years, allowing the implementation of powerful visualization tools at a low cost. They are starting to be widely used in medicine [27,28,29,30,31,32]. Here, we describe the process for implementing these tools and evaluated the results with medical specialists considering the advantages and disadvantages of augmented and virtual reality technologies.

Our main objectives for the designed software system are: easy upload of digital imaging and communication on medicine (dicom) images to the cloud platform, artificial vision module to automatically segment certain anatomical structures, 3D model construction module based on the results of automatic segmentation, and an augmented and virtual reality platform for the visualization and manipulation of 3D models, allowing non-invasive pre-surgical studies. This paper demonstrates the complete system that we developed (named Nextmed) and details one of the algorithms that we designed for the artificial vision module that allows automatic segmentation, in this case, of lungs, showing the results obtained by our lung segmentation algorithm.

The main novelty of this project is the implementation of a complete and modular platform to allow the visualization of medical images with AR and VR, with no customization of the product for each patient, as the whole process is automated. The project has been tested with medical professionals to validate the first version of the platform and to get feedback to improve it.

We first explain how we obtained enough data to start the research, and then we describe the process for developing artificial vision algorithms for automatic segmentation. The different tools used for the implementation of the cloud platform and the visualization tools with AR and VR are analyzed. Then, we describe all the results starting from the dicom processing, showing the results of each stage of the computer vision algorithm for automatic lung segmentation and, finally, we analyze the visualization tools that we designed to allow medical specialists to manipulate the 3D models that were created in the previous steps. We provide an analysis of our results, comparing them with other studies and looking to the future to see how computer vision, artificial intelligence, augmented reality, and virtual reality will be used to improve medical practice. Our conclusions are provided in the final section.

## 2. Materials and Methods

Environment configuration was necessary to start programming (Section 2.1). Then, a web platform to upload dicom files was designed (Section 2.2) and computer vision algorithms were implemented for automatic segmentation (Section 2.3). Once we get a 3D model for segmented area and different files have been saved (Section 2.4), a professional can visualize the result with an AR platform and VR platform (Section 2.5).

### 2.1. Development Environment for Segmentation Module

Python 3.7 was used for applying the algorithm using mainly the Insight Segmentation and Registration Toolkit (ITK) library [33,34]. Jupyter Lab [35] was employed for the graphical interface, with the Itk-widget, which allows the visualization of 3D models using Visualization Toolkit (VTK) in a browser, in Jupyter’s own environment. The distributions available in both the Python Pip and Conda dependency managers do not present some of the wrappers necessary for the realization of our algorithms. Therefore, to use specific modules, such as ITKVtkGlue, we compiled the ITK source code using CMake and we implemented some wrappers to facilitate algorithm implementation.

**Figure 1:**
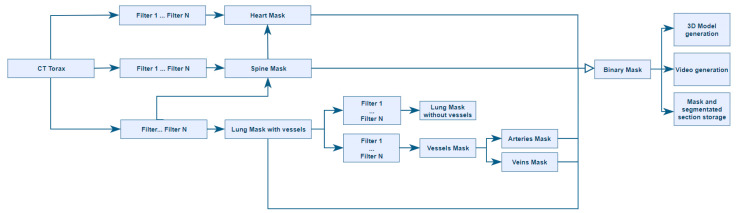
Tree of the different segmentation techniques.

For the ITK algorithms, encapsulations of the functions used were designed similar to those of SimpleItk (library that encapsulates the ITK functions to facilitate their use). As some functions were not available in SimpleItk, we compiled ITK instead of using this wrapper. Therefore, an environment was configured, using the ITK and VTK libraries, for generating a set of artificial vision algorithms to perform automatic segmentation of different anatomical structures, as represented in Figure 1.

### 2.2. Acquisition of Medical Images

To prove that the artificial vision algorithms designed are reliable regardless of the radiological result employed as input, we used a large number of computed tomography (CT) studies during the implementation of our algorithms. Specifically, we used the Lung Image Database Consortium and Image Database Resource Initiative (LIDC-IDRI) dataset so that the algorithms automatically segmented and created a 3D model of each of the results. Seven medical imaging centers and eight companies collaborated to create the LIDC-IDRI dataset, which contains 1018 cases (helical thoracic CT scans) from 1010 patients [36,37].

**Figure 2:**
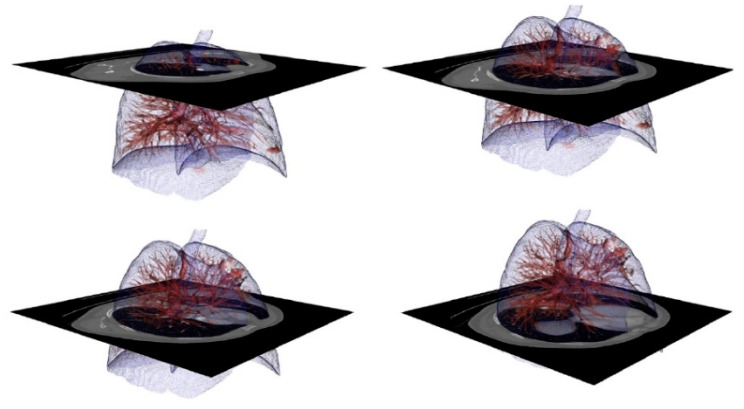
Digital imaging and communication on medicine (dicom) images of the Lung Image Database Consortium and Image Database Resource Initiative (LIDC-IDRI) dataset with the volume superimposed. Visualization used to check segmentation results during implementation. (**A**) Image 20; (**B**) Image 90; (**C**) Image slice 170; (**D**) Image slice 235;

Figure 2 depicts the images generated from one particular study, which, in this case, are merged with a 3D representation in the form of the voxels of these images.

**Figure 3:**
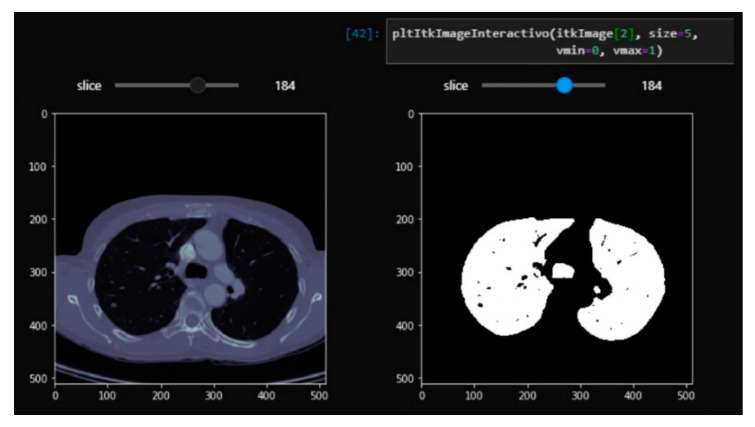
Real-time interactive viewer for the complete tomography using the original image (**left**) and a binary mask (**right**) with our Jupyter Lab’s visualization module.

**Figure 4:**
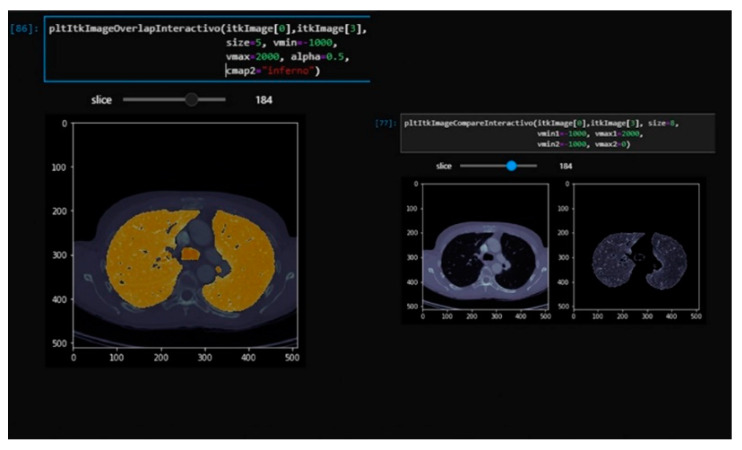
Real-time interactive viewer for the complete tomography with overlapping segmentation and the original image (**left**). The original tomography and segmentation of lung without vascularization is on the **right**. Visualization with our Jupyter Lab’s visualization module.

Using the Jupyter widgets and matplotlib tools, a layered visualization system was developed with a slider so that the layers of the different segmentation steps could be interactively navigated in the notebook. This allows the documentation of the segmentation steps and the detailed visualization of the results of each layer to allow for rapid analysis. This interface is useful for checking the steps of the segmentation and is partially shown in Figure 3 and Figure 4. This development can help researchers in the source code implementation process.

### 2.3. Computer Vision Algorithms

Both ITK and VTK libraries were used to treat the medical images and for their visualization. Our algorithms were designed using the different methods that these libraries provide. Note, these methods do not segment by themselves; they are just computer vision algorithms that must be correctly used to obtain a complete segmentation algorithm.

Some of the methods that have been used for filtering are: BinaryThresholdImage Filter, ThresholdImage Filter, BinaryDilateImage Filter, BinaryErodeImage Filter, BinaryMorphologicalClosingImage Filter, BinaryMorphologicalOpeningImage Filter, and VotingBinaryIterativeHoleFillingImage Filter. Some methods used for noise reduction in medical images are: GradientAnisotropicDiffusionImageFilter and MeanImageFilter. To perform operations with binary images, some of the functions that we use are: AndImageFilter, OrImageFilter, XorImageFilter, and SubtractImageFilter; these methods provide a code to perform the common operations: and, or, xor and subtract. For working with regions, we use: RegionOfInterestImageFilter, ExtractImageFilter, PasteImageFilter, and GetLargestPossibleRegion. We also work with labelmaps to determine the details of groups inside a volume. For example, a group or labelmap could be a lung, stretcher, or a group of voxels that represent some noise in the volume. We use the following methods to work with labelmaps: BinaryImageToShapeLabelMapFilter, ShapeOpeningLabelMapFilter, LabelMapToLabelImageFilter, LabelMapToBinaryImageFilter, GetNthLabelObject, GetNumberOfPixels, GetNumberOfLabelObjects, GetBoundingBox, and RemoveLabelObject (the last five are methods inside the LabelMap class). To apply a mask to a volume to segment a certain area, we use MaskImageFilter and MaskNegatedImageFilter.

Researchers use ITK as an artificial vision library specialized in medical images to build our segmentation algorithms, while VTK allows us to visualize images and volumes [38,39,40,41]. Both libraries have been developed by Kitware (Clifton Park, NY, USA) and are used by many medical imaging programs, such as 3DSlicer or Osirix.

**Figure 5:**
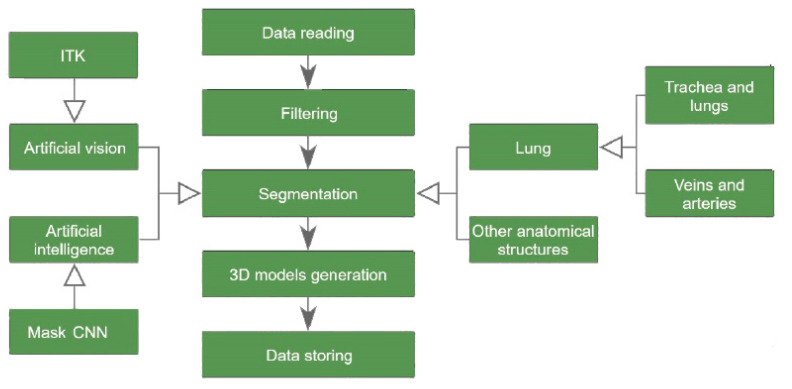
Diagram of the process for automatically generating 3D models.

As shown in the diagram in Figure 5, the designed process begins by reading a set of dicom files from a computed tomography performed on a patient. The process ends with the segmentation results being stored in .nrrd (Nearly Raw Raster Data), .obj, and .webm video formats. A set of alternative data is generated, such as a histogram with the intensities in terms of Hounsfield unit (HU) values that appear in the images.

At this moment, the Nextmed platform segmentation module includes a CNN called Niftynet [17] for the segmentation of spleen, kidney, gallbladder, esophagus, liver, stomach, pancreas, and duodenum. The aim of this project is to include current research for automatic segmentation in the Nextmed segmentation module so that we can use our own algorithms or existing ones.

For the development of vision algorithms, we used different techniques, which can be summarized in the following categories:

- Processed by thresholding: based on keeping the voxels whose intensities are within a fixed range, and setting the rest to a fixed value that is interpreted as the background of the image. This process is useful for separating structures when no different regions with similar intensities exist.
- Morphological processing: based on form, such as erosion and dilation.
- Geometric/positional processing: based on the relative or absolute position of elements.

### 2.4. 3D Reconstruction and Storage

Once the segmentation of an anatomical region is obtained, its binary mask is used to generate a 3D mesh using VTK. To obtain this, we first created an isosurface from the volume using vtkFlyingEdges3D as the main method. After that, we attempted to reduce the polygon size of the mesh using vtkDecimatePro. Then, the number of polygons was reduced and a Laplace smoothing was applied using vtkSmoothPolyDataFilter [42]. This smoothing moves each vertex to the middle position of the adjacent vertices following Equation (1). Thus, a lighter mesh is generated that can be rendered in devices of lesser power and occupies less space on the hard disk.

(1) $$U\left(P\right)=\frac{1}{n}\overset{n}{\underset{i=0}{\sum }}\left(Qi-P\right)$$

where *n* is the number of adjacent vertices to node *P*, and *Qi* is each of those vertices. More explanations of this algorithm are provided in a previous report [42].

In the next step of the process, this mesh is converted into an .obj file that can be printed using a 3D printer, which can be useful, for example, in cases where the creation of prosthesis for a patient is being studied. Next, we generated a video with the segmentation overlapping each layer of the image and going through the layers to quickly check for correct segmentation and for reproducing errors. The video shows the result of the automatic segmentation, which is useful for detecting any possible errors during the developmental phases of the algorithms.

Subsequently, a HU histogram is stored to be used as a guide in the case of any possible problems or for future improvements to the algorithm. This histogram shows the Hounsfield values that were detected in the image.

The next step is saving the segmentation produced in an .nrrd format, which allows storing all layers in the same file, occupying less space than dicom, and being easier to handle due to its reduced number of fields. We maintained the spatial position to show several anatomical regions (from the execution of different segmentation algorithms) in their corresponding spatial positions simultaneously.

### 2.5. 3D Visualization Platforms

Unity3D was used as the graphical engine to develop the different versions of the Nextmed visualization applications. This was achieved using C# language to program all the program logic, as well as development patterns, such as the Model View Controller (MVC), which allow scripts that do not change regardless of the version of the application in question (AR, VR, or personal computer) to be kept together. For the AR version, we used the library provided by Vuforia that allows integration with Unity3D. For the VR version, we used Oculus SDK. In addition, shaders were used, such as multi-slice, for some of the functionalities implemented. Shaders consist of a source code associated with a surface that specifies how the surface should be rendered; in this case, we used it to indicate that one or more parts of a 3D model are not rendered. Web services running on Apache Tomcat and a MySQL database for storing the necessary information were designed to create a cloud platform. However, the cloud platform is being re-developed from scratch to create a highly scalable cloud, using Angular [43] for web development, and Django [44] as the web framework, with OAuth 2.0 authentication [45]. Some of these technologies are used by important organizations such as National Aeronautics and Space Administration (NASA), as they allow the implementation of secure cloud environments. We need this kind of security as we are storing medical data and we want to install this project in hospitals next year.

## 3. Results

### 3.1. From dicom to 3D Models with Automatic Segmentation

The main result of this work is the ability to automatically segment different anatomical regions and to provide tools for visualizing these results in three dimensions, using augmented and virtual reality technologies.

We now focus on the automatic lung segmentation algorithm and examine the results obtained by each of the phases of that algorithm. Whereas other algorithms [46,47] were developed to segment lung nodules, we wanted to obtain the complete mesh of the lungs, thus we focused on obtaining the complete structure without identifying other structures, such as the nodules.

We use the medical images resulting from LIDC-IDRI-0121 to explain the process. Slice number 100 will be shown, unless otherwise indicated, because the contour of the lung is perfectly appreciated in this slice.

**Figure 6:**
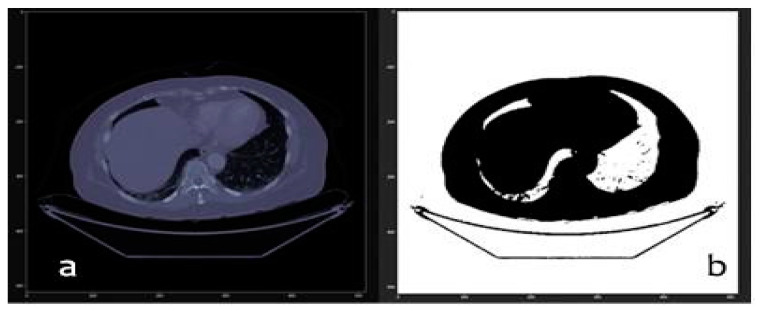
(**a**) Original slice number 100 and (**b**) thresholded slice number 100 of LIDC-IDRI-0121.

First, we have the original dicom image without any type of processing, as shown in Figure 6.

In the next step, we applied a series of filters to remove the edges. We used the Hounsfield units to select only those voxels from the regions in which we were interested, which was between the values of −4000 and −400. The result of this threshold filtering is the mask that is shown in Figure 6b. We then applied morphological filtering algorithms, such as erosion, whose formula is shown in Equations (2) and (3). This consisted of eliminating voxels, by applying an erasure strategy based on the matching of each voxel and its neighbors with a comparison matrix that we call kernel. Those voxels that, together with their neighbors, form a matrix like the kernel, are eliminated. The developed algorithm uses a matrix for the kernel with a spherical shape of radio equal to 10. The result is a three-dimensional mask that we call MASK_01.

*A* Ө *B* = {*z* ∈ *E*|*B_z_* ⊆ *A*} (2)

*B_z_* = {*b* + *z*|*b* ∈ *B*}, ∀*z* ∈ *E* (3)

where we define the erosion of the binary image *A* by the structuring element *B*, where *E* is the Euclidean space or an integer grid, *A* is a binary image in *E*, and *B_z_* is the translation of *B* by vector *z*.

**Figure 7:**
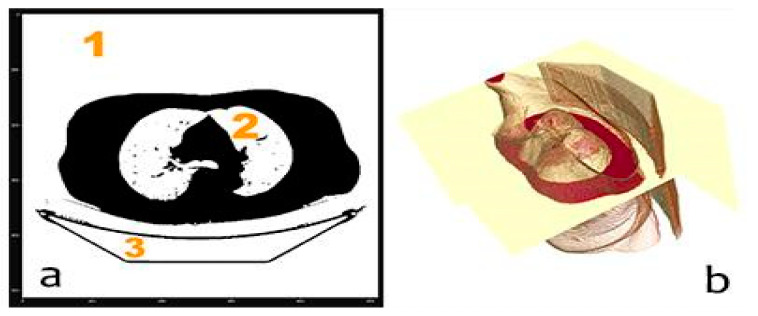
(**a**) 2D non-definitive mask with stretcher and groups of voxels and (**b**) 3D mask volume representation for mask.

We removed the stretcher (group 3 in Figure 7a) only in the first slice by removing fine contours as those that define the stretcher. To do so, we applied a hole filling algorithm using VotingBinaryIterativeHoleFillingImageFilter (ITK). We had to obtain the best values for the parameters of this filter to only remove the stretcher: 700 iterations and a radius of 2 pixels. Although it seems that three groups appear in the mask (Figure 7a), there are only two, as groups 1 and 3 are connected by the first slice (where the stretcher was removed).

**Figure 8:**
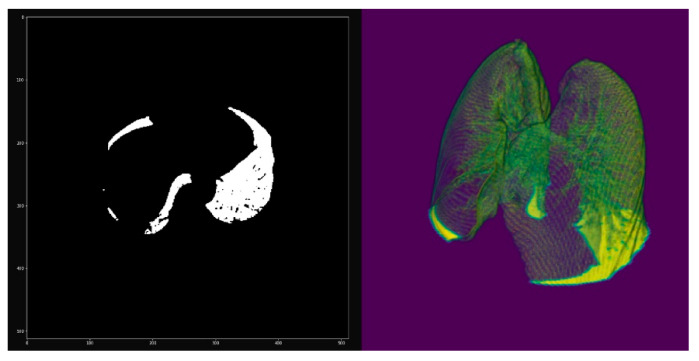
Non-definitive mask without stretcher: 2D slice (**left**) and 3D volume (**right**).

Once the small groups that could appear due to the presence of noise, for instance, are eliminated, the next step is eliminating groups whose dimension in the x- or y-axis coincides with the maximum dimension of the image in the x- and y-axes, respectively. That is, group (1 + 3) is eliminated with only the lungs remaining, obtaining a new 3D mask that we call MASK_02, as shown in Figure 8.

**Figure 9:**
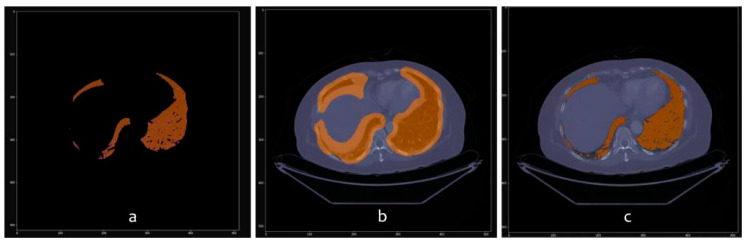
(**a**) Overlap to compare mask before (purple) and after (orange) threshold filtering (left), (**b**) dilation filter (middle), and (**c**) the result of erosion: final mask (right).

Then, MASK_02 was applied to the original image, obtaining the volumetric image RES_01 (Figure 8). However, due to the morphological filtering algorithms applied in the previous steps, such as erosion and dilation, the mask covers an area slightly larger than that of the lung. Thus, we again needed to apply a threshold filter to improve the detail of the edges of the mask, producing the new mask MASK_03. Figure 9 shows how, in the purple pixels, the mask covers a slightly larger area than what is obtained after applying the threshold filtering.

Once we obtained the definitive mask MASK_03, we used this mask to generate the 3D mesh, which can be exported to .obj or .stl format for printing and visualization in the augmented and virtual reality platform. Finally, by applying MASK_03 to the original volume, we obtained a volume with the lung completely segmented.

**Figure 10:**
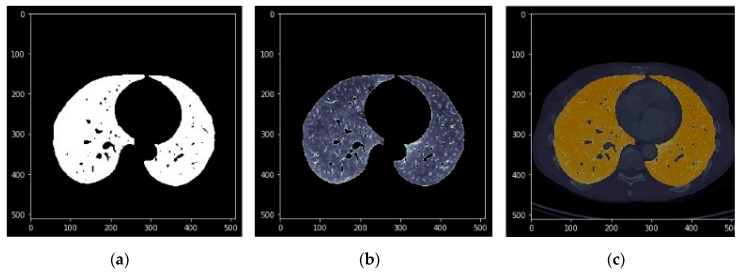
Lung segmentation with veins and arteries. (**a**) Binary mask; (**b**) DICOM with binary mask applied; (**c**) DICOM with binary mask overlapped and highlighted.

**Figure 11:**
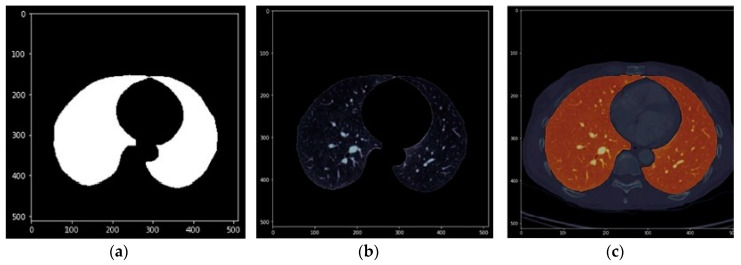
Complete lung segmentation. (**a**): Binary mask; (**b**): DICOM with binary mask applied; (**c**): DICOM with binary mask overlapped and highlighted.

**Figure 12:**
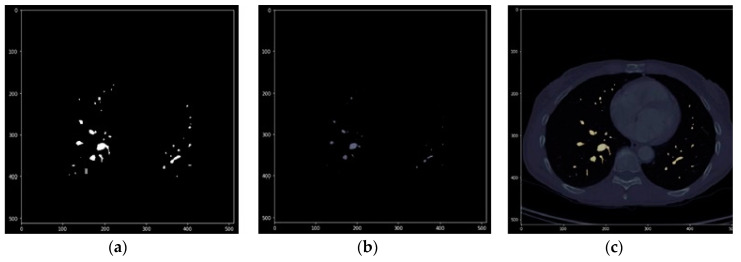
Segmentation of pulmonary veins and arteries. (**a**): Binary mask; (**b**): DICOM with binary mask applied; (**c**): DICOM with binary mask overlapped and highlighted.

The images in Figure 10, Figure 11 and Figure 12 show the segmentations obtained for the pulmonary lungs with and without veins and arteries, as well as for the veins and arteries in isolation.

For the evaluation of the results, we followed different phases. First, the development team reviewed each result to improve the algorithm. Once the algorithms were adjusted and the segmentation had more than 95% success, then all the results were studied by medical professionals of University Hospital of Salamanca (Salamanca, Spain), who evaluated the segmentation process by examining 3D models and the videos generated by the algorithms, studying the Hounsfield histogram to see if the values coincide with those expected for a certain anatomical region. As a result, for the lung algorithm, 98% of the segmentation was correct, which is an excellent result in comparison to other successful algorithms that produce 96% correct segmentations [46]. The only failures were due to scans with a very high level of noise, which caused the resulting 3D model to have some holes on the surface. One lung segmentation algorithm result can be seen in Video S1. We have to highlight that the results derived from other researches were obtained on more consistent numbers of images. For the next phases, we will evaluate results with more images.

**Figure 13:**
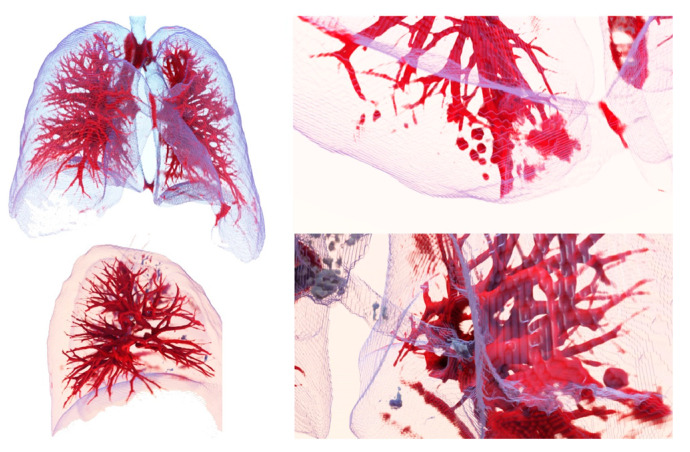
Volume representation of the result of automatic segmentation for lung with blood vessels. **Upper left**: complete lung with veins and arteries. **Upper right**: an enlargement of an area with a detected tumor. **Lower left:** the right lung. **Lower right**: enlarged view of the hilum area.

Figure 13 shows images obtained using the Exposure Render program [48], with several filterings of the segmented region of the lung with veins and arteries. The images correspond to a .mha file obtained as a result of an automatic lung segmentation. The color map that transforms the intensities of the Hounsfield scale into color was manually adjusted, so that veins and arteries can be observed from a larger to a smaller scale.

This filtering can be performed in real time in devices with less processing and rendering capacity than a personal computer (PC), such as virtual reality glasses or smartphones, using augmented reality techniques. This is important, as no specialized workstation is needed and anyone can perform filtering using only a smartphone.

We created a Github repository for this project, where researchers can find the files indicated above, at: https://github.com/arsoft-company/nextmed.

The processing time of the algorithm (lung) per result was an average of two minutes (Table 1 provides some examples). The processor used was an Intel i5 9600K (Inter Corporation, Santa Clara, CA, USA) containing six nuclei that works at a maximum frequency of 4.6 GHz and with an L3 cache memory of 9 MB, accompanied by a RAM memory of 16 GB to 2400 MHz. All the values shown in Table 1 represent the time required to execute the full process: load dicom, segmentation, 3D reconstruction, and saving all the results, including a video with all the images segmented, Hounsfield histogram, .mha file, .nrrd file, and .obj file.

The heart segmentation algorithm has to locate the spine first, which takes about seven minutes. We had to locate the spine to improve the first versions of our algorithm, as the spine also appeared near the heart. So, we segmented the spine and the heart, and then we subtracted to the second from the first result, obtaining only the spine. This is the reason why our heart segmentation algorithm is slow. We have to take into account that approximately 25% of the time is used for 3D reconstruction and 5% is used for data storage. These times also depend on the hardware used.

Since artificial vision algorithms were implemented in this phase for lung segmentation and not artificial intelligence and compute unified device architecture (CUDA) programming was used, it is possible to improve the processing time [49]. CUDA programming is an architecture oriented toward parallel computing, so that large amounts of code can run simultaneously to generate results in parallel.

### 3.2. Results Visualization

**Figure 14:**
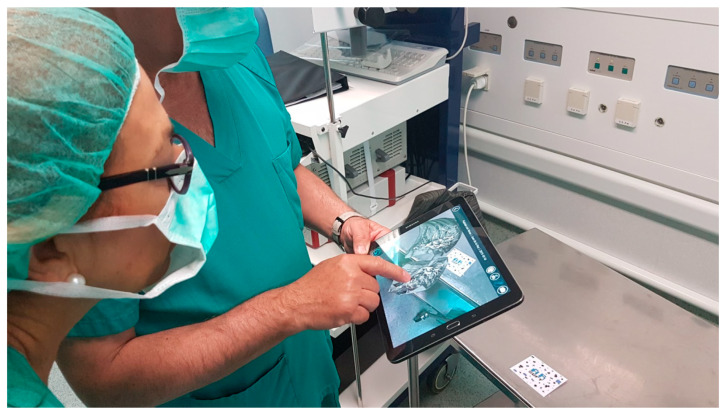
Visualization image of a segmented lung in the augmented reality platform with a transversal cut.

**Figure 15:**
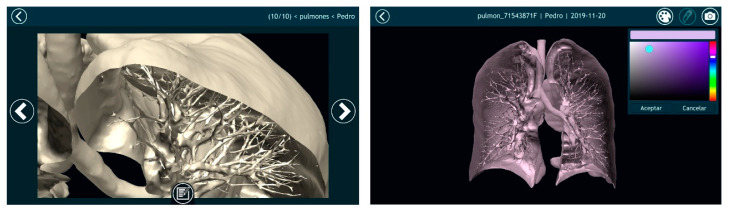
**Left** image shows a screenshot where the professional can add comments that can be accessed later during a surgery. **Right** image shows a cut lung with veins and arteries painted.

**Figure 16:**
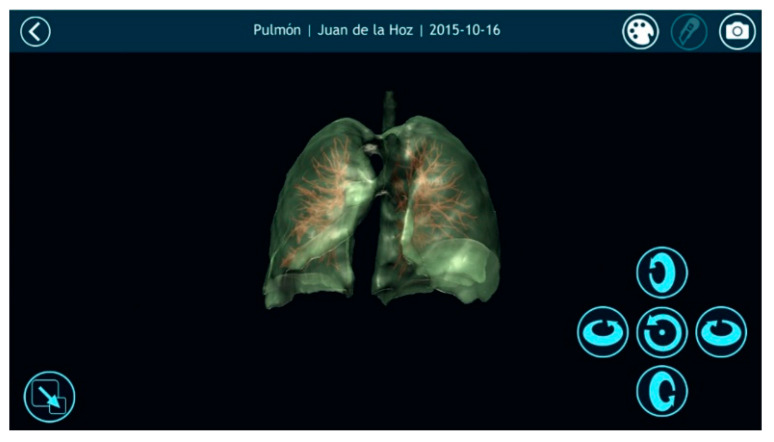
Visualization of a segmented lung with its veins and arteries in the personal computer (PC) version of the visualization platform, with transparency applied to the lung mesh.

Once automatically segmenting an anatomical region was possible and the result of that segmentation was converted into a smooth 3D mesh, the next step was to offer doctors the most appropriate tools for visualizing the 3D model. For this purpose, Nextmed offers three different tools: an augmented reality platform (Figure 14), one based on virtual reality (Figure 15), and a PC version (Figure 16). The first two provide an innovative method for studying radiological results and allow the full potential of the three dimensions to be exploited.

**Figure 17:**
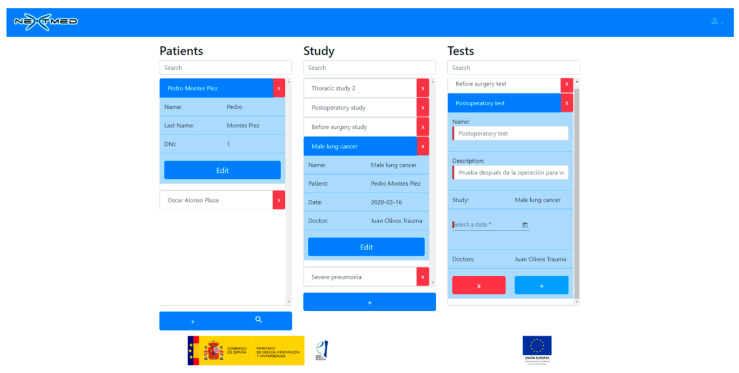
Web application to manage dicom images for different patients (patient data are not real).

**Figure 18:**
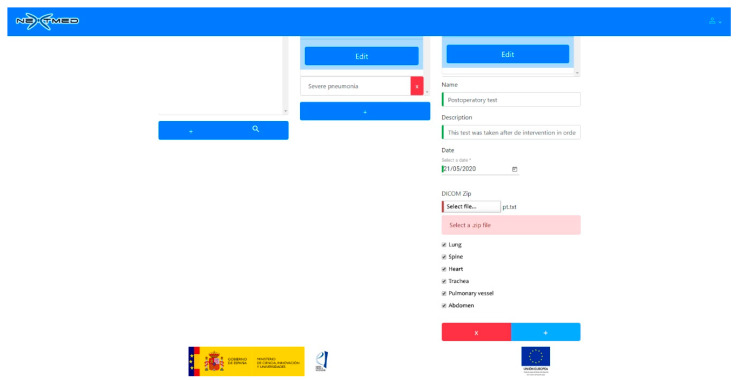
Loading new dicom images to web platform selecting what anatomical regions should be segmented and modeled.

To view a 3D model, the doctor has to upload the dicom images to our cloud platform (Figure 17 and Figure 18). A web application has been developed for this, allowing the doctor to select a patient, a radiological study, and a set of segmentations. Once the dicom images are uploaded, a program will segment them as soon as the CPU is available; as many segmentations can be performed simultaneously, this program must decide what algorithms to run to avoid system errors due to CPU overhead. As soon as the algorithms finish their execution, the generated 3D models will be available for the medical professional to be visualized with augmented and virtual reality.

**Figure 19:**
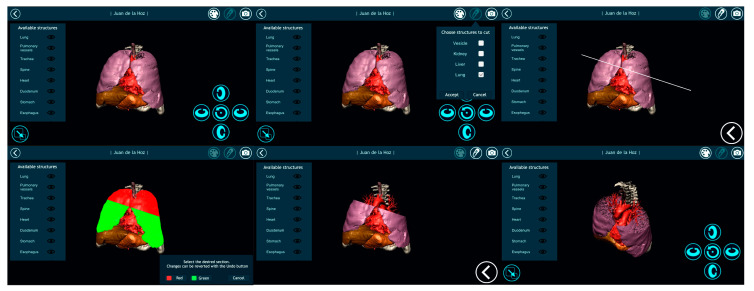
Visualization platform with a cutting sequence for the different anatomical regions of a patient.

In addition, the system offers the medical professional different functionalities to facilitate diagnosis and to complete a pre-surgical study that can later be visualized during the surgery. An example of these features is the multiple cut tool, which allows sections to be created only in certain regions, while keeping the others intact. This is depicted in Figure 19, where different anatomical regions automatically segmented from the same medical image are visualized simultaneously. As it is also possible to capture screenshots and write notes for each screenshot, the medical professional can create resources that can be visualized later during surgery. Even an augmented reality glass can be used during surgery to see the 3D model of the patient and the notes, which is useful, as screen touching is not required to interact with the visualization system due to hands recognition.

## 4. Discussion

The work on the Nextmed project represents a qualitative leap in terms of the study of radiological results, mainly due to two important points. Firstly, we provide the possibility of using automatic segmentation in the daily work of medical professionals, and secondly, we allow the industrialized visualization and manipulation of 3D models (not a specialized method for specific cases) using any computer or mobile device, through the use of augmented and virtual reality.

Until now, systems have been designed to visualize a specific 3D model using these techniques, and many of them are used only for training purposes [50,51,52,53,54,55,56,57]. However, the future of these technologies in the medical sector will be to use them for all radiological results obtained from patients and not only for specific cases. This is precisely the objective of Nextmed: to bring the advantages of augmented and virtual reality to all hospitals, since the tools for doing so are now in place—any computer or phone, as well as augmented and virtual reality glasses.

Therefore, the chief difference of this project from other similar works is that Nextmed has been developed with the idea of using these technologies in a hospital setting and on a daily basis. Whereas other studies [5,6,7,8,9,10,11,12] focused more exclusively on segmentation algorithms, this work includes these algorithms as part of a complete platform that addresses other issues. In relation with previous publications regarding the Nextmed project [32], main novelties are that the system has now been tested in greater detail by professionals from Salamanca Hospital, including radiologists, surgeons, and other specialists, as we wanted to obtain feedback from different points of view. In addition, a cloud platform has been designed and visualization platforms now have new functionalities.

Although many workstations and segmentation programs, such as 3D Sclicer or Osirix, include semi-automatic segmentation techniques, up to three hours may be required to obtain proper lung segmentation. Automatic segmentation therefore provides a considerable improvement, being able to complete the process in just a few minutes or even a few seconds, if using the right hardware. Table 1 lists the times required for our algorithms to produce different radiological results, improving the results of other algorithms [46,47].

The central aspects of this work can be summarized in the following points:

(1) Different anatomical structures can be automatically segmented and a 3D model can be generated in any computer (a workstation is not necessary).
(2) A tool is offered to physicians to visualize medical images in 3D with three different versions: augmented, virtual reality, and computer.
(3) All algorithms were tested using more than 1000 dicom images from computed tomography.
(4) This technology was prepared for its implementation in the daily work of radiologists and specialists, as the entire process is automated.
(5) This work has given rise to the Nextmed project, which is currently still in progress.

### Future Work

In the future, compatibility with nrrd and dicom files in the augmented and virtual display platforms could be considered, which would allow them to interact with volumes of voxels, as well as with meshes. This would involve the introduction of the VTK library in the graphic engine provided by Unity3D, which would allow the voxels of a dicom or nrrd image to be rendered in real time and enhance the doctor’s ability to study a particular region. For example, areas could be manually isolated based on the Hounsfield units using a similar method to traditional programs for processing medical images. To achieve this, OpenGL can be used to render images using VTK inside the Unity3D environment.

Over the coming years, AI will change diagnostic methodology via radiological results. This will allow medical professionals to analyze radiological results and to improve the existing visualization techniques. The use of artificial intelligence to support artificial vision algorithms during automatic segmentation would allow the segmentation processes to learn over time, obtaining increasingly better results. AI also offers the possibility to employ automatic diagnostic techniques. In some cases, the use of AI has already been successful [58], such as detecting metastasis [59], diabetic retinopathy [60], or predicting cardiovascular risk factors [61].

Therefore, the inclusion of these AI techniques will allow the physician to study medical images more efficiently and to take advantage of a first analysis to recognize alterations that could be otherwise overlooked. For example, including a nodule identification module software with AI could be interesting.

## 5. Conclusions

Medical professionals state that non-intrusive access to 3D models through the use of augmented reality glasses during surgery could provide a significant additional advantage over traditional workstations. Notes and images taken during the pre-surgical study are easily accessible, which is of interest to surgeons, who cannot touch anything during surgery.

The noise that appears in many medical images poses a challenge for the artificial vision algorithms at the time of segmenting certain regions. Nevertheless, the devices used to create these images are becoming increasingly precise, which is why the number of cases with a sufficient amount of noise to produce unsatisfactory segmentation results is much lower. In some cases, this is less than 1%, which is why we can say that this does not pose a problem for automatic segmentation.

The application of binary masks has been useful for the development of segmentation algorithms; however, creating masks that perfectly demarcate the regions is difficult. This could be enhanced by applying AI techniques that improve this delimitation based on learning. One of the objectives for which different files are generated during the segmentation process (see Section 2.4) is that, in the future, these files can be used to train a neural network.

Studies have detected variations in the dicom with respect to the 3D mesh, due to the conversion process [57]. Although the generated 3D models faithfully reflect the results visible in the medical images, the minimization of this error must be considered a priority, since precision is a fundamental factor.

In the future, doctors and radiologists will use augmented and virtual reality to study medical images on a routine and daily basis. However, to achieve this, a 3D model of the anatomical area of interest must be affordably generated. As such, we must continue to progress the implementation of automatic segmentation techniques that may be applicable for all types of cases. In this study, we also focused on medical images obtained from computed tomography, but it is necessary to continue with research that includes compatibility with magnetic resonance.

## Figures and Tables

**Table 1:** Execution times for different anatomical structures, including segmentation, reconstruction, and results storage.

CT Scan	Region Segmented	Execution Time	Image Resolution	Number of Slices
**LIDC-IDRI-0001**	lungs	6.94 s	512 × 512	133
	lung vessels	1 min 23 s		
	trachea	11.4 s		
	spine	1 min 42 s		
	heart	2 min 18 s		
**LIDC-IDRI-0002**	lungs	13.3 s	512 × 512	261
	lung vessels	1 min 56 s		
	trachea	31 s		
	spine	3 min 25 s		
	heart	4 min 40 s		
**LIDC-IDRI-1004**	lungs	25.3 s	512 × 512	529
	lung vessels	3 min 16 s		
	trachea	1 min 28 s		
	spine	6 min 54 s		
	heart	10 min 2 s		

## Supplementary Materials

The following are available online at https://github.com/arsoft-company/nextmed. Video S1: Arsoft_AutomaticLungSegmentation.mp4, and other related resources.
